# Psychosocial drivers influencing local food purchasing: beyond availability, the importance of trust in farmers

**DOI:** 10.3389/fnut.2023.1204732

**Published:** 2023-09-29

**Authors:** Valentina Carfora, Patrizia Catellani

**Affiliations:** Department of Psychology, Faculty of Political and Social Sciences, Catholic University of the Sacred Heart, Milan, Italy

**Keywords:** local food, food availability, trust in farmers, food attribute, COVID-19

## Abstract

**Introduction:**

Although consumers bought more local food during the changing context of pandemic COVID -19, this positive modification may not become a stable habit afterward.

**Methods:**

To understand this change in drivers of consumers' intention to buy local food, we investigated the role of perceptions of various intrinsic and extrinsic attributes of local food, its perceived quality, price and availability, and consumers' trust in local food producers. We also investigated the role of sociodemographic variables as well as the moderating role of consumers' stage of change (i.e., absence, reduction, maintenance, and increase) in the purchase of local food.

**Results:**

Structural equation modeling results on a representative sample of Italian consumers (*N* = 511) showed that local food availability is the main driver of purchase intention (*β* = 0.20; *p* = 0.001), especially among consumers who have changed their habits toward buying local food (reduction stage = *β* = 0.24; increase stage = 0.30; *p* = 0.001). In addition, trust in local food producers was found to be a key antecedent to consumers' perceptions of local food as environmentally friendly (*β* = 0.57; *p* = 0.001), healthy (*β* = 0.55; *p* = 0.001), authentic (*β* = 0.58; *p* = 0.001), tasty (*β* = 0.52; *p* = 0.001), socially sustainable (*β* = 0.59; *p* = 0.001), and as a product with a good appearance (*β* = 0.55; *p* = 0.001).

**Discussion:**

Overall, these results improve our understanding of which food attributes should be emphasized in communication to promote the purchase of local food.

## 1. Introduction

Recent crises have brought the vulnerability of global food supply chains to the fore (1) and demonstrated that the availability of several food categories may be at serious risk. These events raise concerns about the resilience of supply chain systems to shocks, i.e., their ability to continuously change and adapt in response to stressors and societal challenges (2). As a result, interest in local and regional food production is increasing significantly and is likely to grow further.

Although there are different definitions of “local food,” in most cases, it is defined as food grown near the consumer (3). In the European context, the Joint Research Centre of the European Commission defines local food as food that is produced, processed, and sold in a specific geographical area, for example, within a radius of 20 to 100 km (4). In the present study, we have referred to local food, taking into account not only geographical proximity but also the sustainable production/distribution methods used by local farmers who do not follow the large-scale distribution logic.

Although consumers purchased more locally produced food during the last years of the COVID-19 pandemic (5), these changes may not become a stable shopping habit after the pandemic (6), e.g., because localized and small food supply chains are less cost-efficient than large retailers and offer less product variety at higher prices (7). A deeper understanding of why many consumers reduced their purchases of local food can be gained by looking at consumers' perceptions of the intrinsic and extrinsic attributes of local food. While intrinsic attributes are the characteristics of the food product itself, such as taste and health, extrinsic attributes are the characteristics that belong to the food product but are not part of it, such as its environmental impact or its expected price availability (8). While there are some studies on pandemic-related changes in consumers' perceptions of the intrinsic and extrinsic qualities of local food (8), there is so far a lack of studies that address what happens afterward.

To fill this gap, the present study aimed to investigate how consumers' perceptions of local food after the COVID-19 pandemic influenced their intention to purchase such food. Specifically, we investigated the role of perceptions of various intrinsic and extrinsic attributes of local food, its perceived quality, price, and availability, and consumers' trust in local food producers. We also investigated the role of sociodemographic variables, as well as the moderating role of consumers' stages of change in the purchase of local food (i.e., absence, reduction, maintenance, and increase).

## 2. Theoretical background

Researchers from diverse backgrounds have studied how different values, beliefs, and attitudes influence consumer preferences for different food products (9). In the case of local food, these key factors are diverse and relate to both intrinsic and extrinsic food attributes (10). Moreover, most scholars pointed out that price consciousness (11), trust (12), and availability (13) play an important role in consumers' decisions to buy local food. In the following, we summarize the theoretical considerations that led us to formulate our research hypotheses and develop the model that we subsequently tested using structural equation modeling. A summary representation of our model and hypotheses can be found in Supplementary Figure 1.

### 2.1. Consumer's perception of local food

The overall evaluation of the quality of local food is an important factor in consumers' intention to buy food from local producers (14). If consumers perceive a product to be of high quality, they may have a higher purchase intention. In contrast, a poor quality perception may lead to a lower purchase intention.

**H1**. Perceived quality of local food increases future intention to buy it.

The overall evaluation of the high quality of local food is shaped by the perception of its intrinsic and extrinsic attributes (15). In terms of intrinsic attributes, consumers tend to perceive local food as a high-quality product due to its sensory appeal, especially in terms of appearance (such as freshness) and taste (16). In addition, local food is perceived as healthier by consumers (10), as their producers often offer information about their production, e.g., what kind of chemicals they use in production (17). Another important characteristic of local food is its perceived authenticity, which is related to aspects such as continuity, credibility, and symbolism of the local agri-food sector (18).

**H2**. A high perception of local food as a product with a good appearance (**H2a**), tasty (**H2b**), authentic (**H2c**), and healthy (**H2d**) predicts perceiving it as a high-quality product.

The same intrinsic and extrinsic characteristics of local food are likely to directly influence consumers' intention to buy local food as well as their actual purchase. For example, previous research has shown that those who perceive local food as safe, clean, and fresh are more likely to buy it (19).

**H3**. A high perception of local food as a product with a good appearance (**H3a**), tasty (**H3b**), authentic (**H3c**), and healthy (**H3d**) increases consumers' future intention to buy it.

Consumers buy local food not only for its perceived sensory and health attributes but also because it contributes to sustainable practices for both the environment and society (13, 20, 21). Indeed, some scientists agree that local food supply chains are produced in a non-industrial, non-mass, and environmentally friendly way and can, therefore, have a low impact on the environment. They reduce greenhouse gas emissions associated with food transport and adopt more ecological practices (e.g., crop rotation, creation of field margins as a retreat for native biodiversity, reduction of packaging, or moderate use of fertilizers and chemicals) (22).

**H4**. A high perception of local food as respectful of the environment (**H4a**) and socially sustainable (**H4b**) increases consumers' perception of the quality of local food **(H5a)** and their future intention to buy it (**H5b**).

Local food purchases are not only influenced by objective price but also by price consciousness (11), i.e., the meaning attributed to objective price and its translation into a more personal or psychological price. Therefore, we expected the perceived price of local food to be influenced by its perceived quality. However, price expectancy is often a barrier to consumers' intention to buy local food (10, 11). When people are very aware of the price of a product, they are generally less likely to choose that product [e.g., (23)].

**H6**. The perceived quality of local food increases its expected price (**H6a**).

The expectation of a high price for local food predicts a low future intention to buy it (**H6b**).

### 2.2. Local food availability

To date, few studies have examined contextual and extrinsic factors that act as barriers to local food choices. Among these barriers, availability plays an important role. When a product is highly available, consumers usually have the intention to buy it (24, 25). In turn, lack of availability is the major barrier to consuming local food (13).

**H7**. The perceived availability of local food predicts consumers' intention to buy it.

### 2.3. Trust in local food producers

Trust is a complex concept that has attracted the interest of many researchers, so much so that it is now often considered one of the key variables in the consumer decision-making process. In particular, due to numerous food scandals and the ongoing industrialization and globalization of food chains, consumer skepticism about food quality and safety has increased in recent decades (26). Certifications and labeling of products or processes usually solve this problem successfully, even though some relevant properties of food cannot be easily certified because they pass through a short supply chain. The credibility attributed to local food producers is, therefore, often based on the assurance that they have the know-how and skills required for efficient and traceable production. Interestingly, trust provides a solution to situations characterized by increasing complexity and a lack of knowledge, as in the case of consumer trust in food and buyer–seller relationships (12). Moreover, previous studies in the field of correlational studies investigating consumer food purchase intention have shown that consumer trust has both a direct and an indirect influence on food purchase intention (27).

**H8**. Consumers' trust in local food producers predicts the perception of local food as having a good appearance (**H8a**), being tasty (**H8b**), authentic (**H8c**), healthy (**H8d**), respectful of the environment (**H8e**), and socially sustainable (**H8f**). Moreover, trust in local food producers predicts the perceived quality (**H8g**) and expected price of local food (**H8h**). Finally, trust in local food producers predicts consumers' future intention to buy local foods, both directly and indirectly (**H8i**).

### 2.4. Sociodemographic variables

Several previous studies have examined the role of some sociodemographic characteristics as important predictors of food consumption [e.g., Winterstein and Habisch (28); Witzling and Shaw (29)], as well as during the COVID-19 pandemic (30, 31). In terms of perceived quality and price consciousness, past researchers have observed the influence of income, age, education, and gender. For example, women and educated consumers with high incomes were found to be more likely to purchase local food (32). In addition, households that lost income reported being more willing to continue the positive changes they had made in their food habits during post-pandemic COVID-19 (31). Based on the above studies, we hypothesized that sociodemographic characteristics would influence beliefs and intentions toward local food. However, we did not develop a specific hypothesis about the relationship between these variables and the psychosocial drivers presented in the sections above, but only two research questions.

**RQ1**: Do age, sex, and education influence the paths linking the psychosocial antecedents of local food choice and the intention to buy local food?
**RQ2:** Does the family's economic condition influence the perception of the price and availability of local food and the intention to buy it?

### 2.5. Stage of change in local food consumption

Some previous studies investigated the impact of perceived local food attributes, price, and quality on its consumption during the COVID-19 lockdown period (21, 33). For example, a study considered the frequency of purchases from short food supply chains during COVID-19 as correlated with diverse psychosocial antecedents, such as environmental perception, perceived food safety, and healthiness (34). However, changes in local food purchasing during the COVID-19 period might also modulate the impact of psychosocial antecedents on future intention to purchase local food. Indeed, a large number of studies have already shown how past behavior and the actual stage of change moderate the effect of multiple predictors of food choices on future intention (35–37). To date, no study has conducted a similar investigation regarding the purchase of local food. In addressing this issue, in the present study, we assumed that the COVID-19 period was an important turning point in consumer behavior, if only in terms of the importance attributed to the issue of health protection. Therefore, we decided to investigate whether the influence of the psychosocial and sociodemographic factors would vary according to what the person did before the COVID-19 pandemic, categorized in terms of absence, reduction, maintenance, or increase compared to pre-pandemic levels.

**RQ3:** Is the impact of local food perceptions (i.e., extrinsic and intrinsic attributes, quality, price, availability, and trust) on future purchasing intention moderated by the consumer's behavior before the surge of the pandemic?

## 3. Materials and methods

### 3.1. Sample and procedure

This study was part of a research project funded by the Catholic University of the Sacred Heart (Milan, Italy) aimed at understanding changes in Italians' behavior after COVID-19. Italy is an exemplary country due to its leading food industry, and sustainable agriculture and is the first in the world for food quality certification (38). The survey data were collected from Italian consumers 1 year after the second wave of COVID-19 closures in March 2021.

The research was conducted according to the rules of the 1975 Declaration of Helsinki, which was revised in 2013. As this research was a non-interventional study (i.e., a survey), we did not require ethical approval. The study was explained to the participants in the online questionnaire. They were informed that they would participate in the survey using their personal computer and that all data would be de-identified and reported only in aggregate form. All participants signed an informed consent form to participate in the study. All participants were fully informed that their anonymity was guaranteed, why the study was being conducted, how their data would be used, and that there was no risk associated with their participation.

In June 2021, a nationally representative survey was conducted in Italy using the Computer-Assisted Web Interview (CAWI) method. The survey was conducted by Ipsos, one of the leading market research companies in Italy. The sample consisted of four stratified and random subsamples representative of an Italian consumer panel in terms of gender, geographic residence, age group, educational attainment, and employment status (*N* = 511; age: mean = 49.46, standard deviation = 16.52, range = 18–90; Table 1). The sample was balanced in terms of gender (men = 268, women = 243). Most participants were highly educated: 40.4% of them had completed high school, and 21.4% had higher education. Most participants were married (62.8%), and almost half of them had a job (49.7%). Before data collection, participants gave their written consent.

**Table 1:** Demographics of the study sample.

**Main sociodemographic characteristics**	**Total sample**
**Gender**	
Female	52.7%
Male	47.3%
**Age**	
18–24 years	8.6%
25–34 years	13.5%
35–44 years	16.0%
45–54 years	21.5%
55–64 years	18.0%
65–74+ years	22.3%
M	49.46
SD	16.52
**Education**	
Secondary school	38.2%
High school diploma	40.4%
University degree	21.4%
**Marital status**	
Single	27.8%
Married/cohabiting couple	61.8%
Separated/divorced	7.2%
Not declared	3.3%

### 3.2. Measures

The online questionnaire was organized into diverse sections that covered different areas of Italians' behavior after the surge of the COVID-19 pandemic. Below, we report the measures relevant to the current study.

First, we asked participants to indicate their age, sex level of education, and family's economic condition.

#### 3.2.1. Stage of change

Then, participants were asked to report their local food consumption during the ongoing year by selecting one of the four options: (1) “I never bought local food,” (2) “I bought local food less than before the surge of the COVID-19 pandemic,” (3) “I bought local food as before the COVID-19,”and (4) “I bought local food more than before the COVID-19.” Statement 1 was coded as the absence stage, statement 2 was coded as the reduction stage, statement 3 was coded as the maintenance stage, and statement 4 was coded as the increase stage.

Next, we invited participants to complete a series of scales on a Likert scale aimed at measuring their perceptions about local food.

#### 3.2.2. Local food attributes

Seven items to measure perceptions about the attributes of local foods [adapted from Ghali-Zinoubi (33); Denver et al. (39); Soonsan et al. (40)], defined as foods produced within 70 km from the place of sale by adopting sustainable methods of production and distribution. Specifically, we asked participants to rate local food *appearance* (“Local food looks nice”), *taste* (“Local food is tasty”), *authenticity* (“Local food is authentic”), *healthiness* (“Local food is healthy”), *environment respect* (“Local food is produced in an environmentally friendly way”), *social sustainability* (“Local food is produced in a way that respects workers' rights”), *quality* (“Local food is high-quality”), and *price* (“Local food is expensive”).

#### 3.2.3. Local food availability

The consumers' perception of local food availability was measured with three items (e.g., “Local food is easy to find”). *α* = 0.87, composite reliability = 0.92, and AVE = 0.80.

#### 3.2.4. Trust in local food producers

The consumers' trust in local food producers was measured with three items (e.g., “Local food producers work according to strict and controlled standards”). *α* = 0.88, composite reliability = 0.92, AVE = 0.79.

#### 3.2.5. Intention to buy local food

This dimension was measured with three items (e.g., “I intend to buy local food in the near future”). *α* = 0.96, composite reliability = 0.97, and AVE = 0.93.

### 3.3. Data analysis

We ran all analyses using MPLUS 7. As preliminary analyses, we tested the measurement model with confirmatory factor analysis. We verified the internal consistency among the observed variables using Cronbach's alpha and composite reliability. We then tested the convergent and discriminant validities of our data using average variance extracted (AVE) values.

Then, we verified our hypotheses and research questions by testing the goodness-of-fit of four nested SEM models. We compared the nested models with the chi-squared difference test (Δχ2). Model 1 tested our H1–H4 about the role of local food attributes. Model 2 tested our H5 by including the paths from local food availability to intention. Model 3 tested our H6 related to the inclusion of trust in local food producers (H5). Model 4 tested our RQ1 and RQ2 related to the inclusion of sociodemographic variables by including age, sex, education, and family economic resources. To verify if local food purchasing after COVID-19 moderated the relationship between antecedents and the future intention to buy local food (RQ3), we conducted a multigroup SEM analysis (41). Then, to disconfirm the invariance of the paths among the study variables across the above groups, we constrained the paths of each group to be invariant in the other groups, and next, we used Wald tests to disconfirm the invariance of the paths.

In all the above analyses, the goodness-of-fit of all models was tested using chi-square and incremental goodness-of-fit indices: root means square error of approximation (RMSEA) < 0.05, comparative fit index (CFI) < 0.90, Tucker–Lewis index (TLI) < 0.90, and standardized root mean squared residual (SRMR) < 0.08 (42).

## 4. Results

### 4.1. Preliminary analyses

A confirmatory factor analysis showed that the measurement model fitted the data satisfactorily (χ*2* (103) = 264.21, *p* = 0.001; *RMSEA*= 0.05, *CFI* = 0.98, *TLI* = 0.95, *SRMR* = 0.03). The standardized item loadings of all study variables varied from 0.71 to 0.94. Composite reliability values were all greater than the minimum threshold of 0.60. Thus, we confirmed the reliability of the measurement model. The standardized factor loadings and the AVE values were all above the recommended threshold (43), showing that all constructs had high convergent validity. Finally, all AVEs were higher than correlations between latent constructs, confirming the discriminant validity of the study variables (43). Table 2 reports the means, standard deviations, and correlations between study variables.

**Table 2:** Means, standard deviations, and correlations between study variables.

**Study variables**	***M***	***SD***	**1**.	**2**.	**3**.	**4**.	**5**.	**6**.	**7**.	**8**.	**9**.	**10**.	**11**.	**12**.
1. Appearance	4.60	1.29	1											
2. Taste	5.00	1.27	0.63^*^	1										
3. Authenticity	5.08	1.29	0.54^*^	0.68^*^	1									
4. Healthiness	5.00	1.29	0.59^*^	0.74^*^	0.76^*^	1								
5. Environment Respect	5.13	1.23	0.45^*^	0.58^*^	0.64^*^	0.66^*^	1							
6. Social Sustainability	5.04	1.25	0.51^*^	0.51^*^	0.58^*^	0.57^*^	0.64^*^	1						
7. Price	4.56	1.28	0.22^*^	0.24^*^	0.16^*^	0.24^*^	0.17^*^	0.17^*^	1					
8. Quality	4.79	1.37	0.58^*^	0.74^*^	0.77^*^	0.74^*^	0.63^*^	0.55^*^	0.21^*^	1				
9. Local food availability	5.04	1.25	0.43^*^	0.47^*^	0.47^*^	0.43^*^	0.38^*^	0.40^*^	0.00	0.45^*^	1			
10. Trust in Local Food Producers	4.58	1.09	0.58^*^	0.56^*^	0.61^*^	0.60^*^	0.55^*^	0.63^*^	0.15^*^	0.59^*^	0.53^*^	1		
11. Stage of change	1.61	1.01	0.21^*^	0.24^*^	0.26^*^	0.27^*^	0.26^*^	0.25^*^	0.00	0.24^*^	0.35^*^	0.21^*^	1	
12. Intention to buy	4.79	1.47	0.37^*^	0.50^*^	0.55^*^	0.55^*^	0.52^*^	0.44^*^	0.02	0.54^*^	0.47^*^	0.49^*^	0.37^*^	1

*M*, mean; *SD*, standard deviation.

* *p* = 0.001.

Results showed that participants recognized authenticity as the main intrinsic attribute of local food and environmental respect as the main extrinsic attribute. Overall, participants perceived local food as available and reported a medium level of trust in local food producers and an intention to buy local food. Importantly, 24.1% of respondents never bought local foods (i.e., absence stage), 11.7% bought less local food than before the COVID-19 (i.e., reduction stage), 46,4% bought local food as before the COVID-19 (i.e., maintenance stage), and 17,8% bought more local food than before the COVID-19 (i.e., increase stage).

### 4.2. Model comparisons

**Figure 1:**
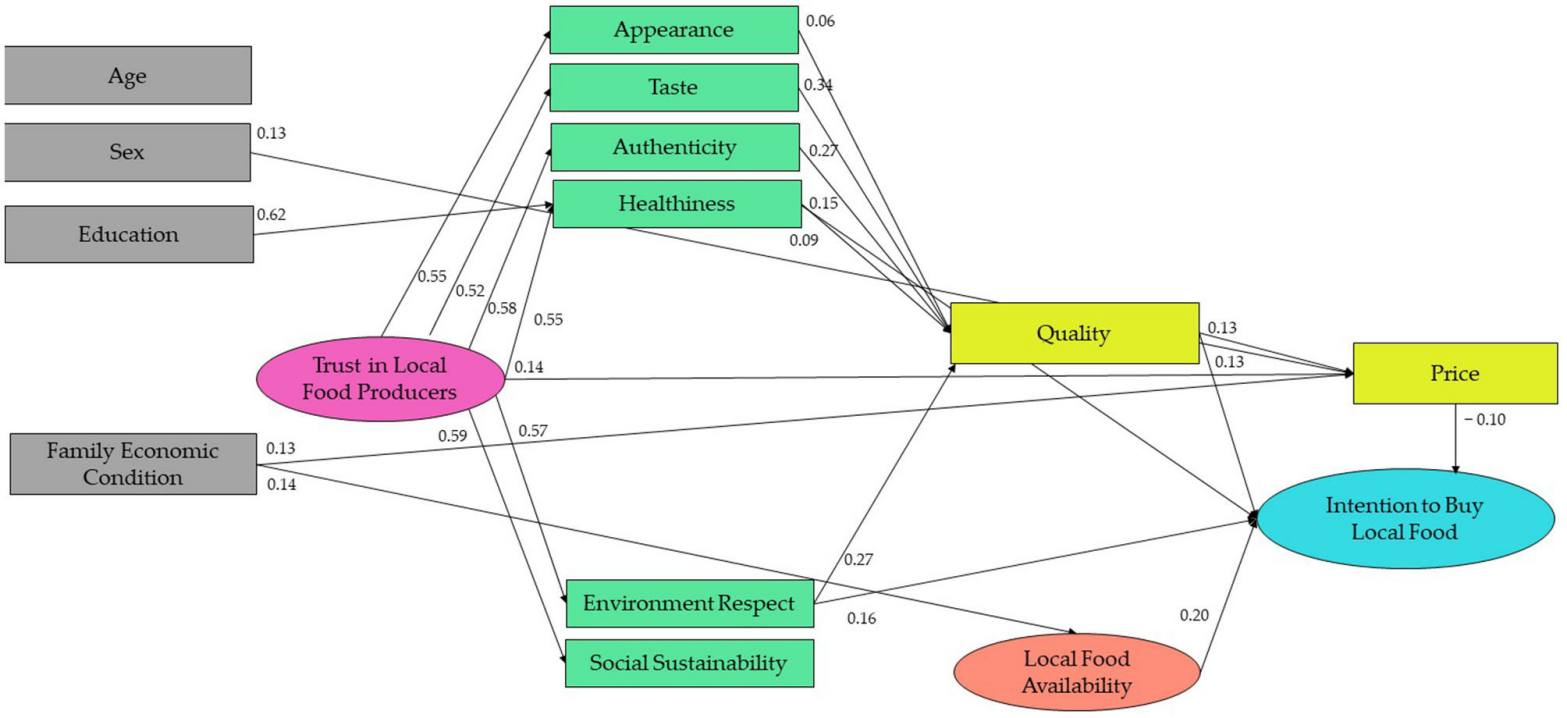
Results of the integrated model to explain the consumers' intention to buy local food.

The results of the comparisons among the four nested models showed that only Model 4 (i.e., the model including intrinsic and extrinsic*α* attributes, quality, local food availability, trust in local food producers, intention to buy local food, age, sex, education, and family economic condition) had acceptable goodness-of-fit (χ*2* = 13.76, *p* = 0.08; RMSEA = 0.04; CFI = 0.99; TLI = 0.98; SRMR = 0.01). The comparison between Models 1 and 2 supported the addition of local food availability (Δχ*2* (9) = 214.45, *p* = 0.001). The comparison between Models 1 and 3 (Δχ*2* (19) = 610.99, *p* = 0.001) and between Models 2 and 3 (Δχ*2* (11) = 396.70, *p* = 0.001) confirmed the opportunity to include trust in the model. Finally, the comparison between Models 1 and 4, Models 3 and 4, Model 3, and Model 4 supported the inclusion of control variables (age, sex, education, and family economic condition, Δχ*2* (46) = 151.83, *p* = 0.001). As expected, the more comprehensive Model 4 was the model that best predicted participants' intention to buy local food (Figure 1). Supplementary Table 1 shows the goodness-of-fit and the standardized coefficients of each tested model.

The study confirmed H1 and H2 that consumers' perception of local food as a high-quality product positively influenced their intention to buy it and that this perception was largely predicted by healthiness, taste, and authenticity, but not appearance. Healthiness had a direct and indirect effect on intention, while taste and authenticity only had an indirect effect through quality perception. Appearance did not have any significant effect on intention. Thus, we confirmed H3b, H3c, and H3d but not H3a. Then, we confirmed H4a and H5a that environmental respect determined the perceived quality of local food and directly influenced the consumers' intention to buy it. Differently, social sustainability influenced neither quality nor intention, disconfirming our H4b and H5b.

As expected (H6a, H6b), consumers' perceived quality of local food increased their expected price. In turn, the consumers' perception of local food as expensive decreased their future intention to buy it. This effect of price on intention was also indirect through consumers' perception of quality. Interestingly, taste and authenticity had a slightly indirect effect on intention via quality and then price, showing that they predicted consumers' intention to buy local food only when it was considered a high-quality inexpensive product.

As regards the contribution of local food availability, it was the most relevant factor in explaining purchasing intention (H7).

As for trust in local farmers, we confirmed that this variable predicted consumers' perception of intrinsic and extrinsic attributes (H8a–H8f), but not perceived quality. Thus, we disconfirmed H8g. Trust in local food producers only indirectly affected consumer intention through health, environment, and price. Higher trust not only led to the perception of local food as authentic and high-quality but also as expensive, decreasing the intention to buy (partially confirming H6i). Therefore, H6i was partially confirmed.

Analyzing the role of sociodemographic variables (RQ1 and RQ2), we found that females perceived local food as more expensive than males, which reduced their intention to buy it. Low-income individuals perceived local food as less available and thus intended to buy it less. Higher education predicted trust in local food producers, which led to greater environmental respect and the intention to buy local food. Consumers' age and education positively correlated with their intention to buy local foods.

### 4.3. Comparison of the integrated model across consumers' stages of change related to local food consumption

The multigroup models obtained an acceptable fit (χ*2* = 63.73, *df* = 32; χ*2* absence stage = 21.62; χ*2* reduction stage = 21.24; χ*2* maintenance stage = 14.10; χ*2 i*ncrease stage = 6.70; RMSEA = 0.06; CFI = 0.99; TLI = 0.90; SRMR = 0.02). Supplementary Tables 2, 3 in report the findings of the Wald test for each comparison used to disconfirm the invariance of the paths among study variables across the four groups of consumers' stages of change. In these analyses, we ran the Wald test only when a path was significant in at least one group.

Compared to those in the reduction stage, people in the absence stage perceived local food as high in quality when also evaluating it as healthy (Supplementary Figure 2). If they trusted local food producers, they perceived local food as tastier and healthier but more expensive. They had a lower future intention of buying local food when they perceived it as expensive. First, compared with those in the maintenance stage, they perceived local foods as tastier, more socially sustainable, and more expensive. Second, women had a greater perception of local foods as environmentally respectful than men. Third, people who had low family economic conditions perceived local food as more expensive. Finally, compared to those who increased local food purchasing after COVID-19, they perceived local food as more expensive when having low family economic conditions, and had lower future intention to buy local food when perceiving it as expensive.

Compared to what we observed in all other stages, people who reduced local food purchasing after COVID-19 perceived it as high in quality, mostly when evaluating local food as authentic (Supplementary Figure 3). Moreover, they intended to buy more local food in the future when they perceived it as healthy. Men strongly perceived local food as more high-quality than women. They also perceived local food as more socially sustainable than those in the increase stage and intended to buy local food more than those in the maintenance stage, when they also perceived local food as a product with a good appearance.

Compared to what we observed in all other stages, in the maintenance stage (Supplementary Figure 4), consumers intended to buy more local foods when they were old. However, they had lower intention when perceiving local food as expensive. Differently from what we observed in the reduction and increase stages, they perceived more local food as healthy and authentic and then evaluated it as higher in quality. In turn, they had a higher intention to buy local food in the future. Interestingly, these effects on future intention were also driven by consumers' trust in local food producers. In addition, when these consumers trusted local food producers, they also perceived local food as tastier and healthier, compared to those who were in the reduction stage.

Differently from what we found in all other stages, a positive evaluation of local food in terms of taste is a strong determinant of the perceived quality in people who increased local food purchasing after the COVID-19 period (Supplementary Figure 5). Moreover, authenticity was not relevant to quality. In addition, they perceived it as more expensive when they had a higher level of education. They also perceived local food as being more available and thus intended to buy local food in this case, when they were old. Compared to those who never bought local food (i.e., the absence stage), they intended to do it in the future when perceiving local food as available. Compared to consumers in the reduction stage, they perceived local food as higher in quality when they also evaluated it as healthy. If these consumers trusted local food producers, they also perceived local food as tastier and healthier. In addition, they expected local food to be more expensive because it is higher quality. This last difference also emerged in the case of the comparison of the increase stage to the maintenance stage.

## 5. Discussion

The study analyzed the psychosocial drivers that influence Italian consumers' intention to buy local food, considering changes in their behavior due to the COVID-19 pandemic. The study found that consumers' perceptions of intrinsic and extrinsic attributes of local food, their expectations of its quality, price, and availability, and their trust in local food producers predicted their future intention to buy local food. The consumers' sociodemographic characteristics also played a role in the model, given that females perceived local food as more expensive than males, which reduced their intention to buy it. In this regard, it appears that there are varying findings in different studies regarding women's preferences and price sensitivity. For example, some studies suggest that Australian and New Yorker women prioritize price attributes more than men (44, 45), while other research indicates that women are more likely to purchase organic food, suggesting lower price sensitivity (46). These differing results highlight the complexity of consumer behavior and the need for further investigation.

### 5.1. The major role of local food availability

The most important predictor of future intention to buy local food turned out to be the availability of that food. While studies of consumers' food choices before the COVID-19 pandemic have shown an important, but not as central, role in food availability and perceptions of control over food purchases (24), the present study suggests that the experience of lockdown restrictions increased the perceived importance of this factor. Local food availability in Italy plays a crucial role in shaping consumer behavior and food choices. The country has a rich tradition of local culinary diversity and offers different diverse options for buying local food. For example, farmer's markets and open-air markets are common, offering an array of locally grown produce, artisanal products, and traditional specialties. Moreover, Italy is renowned for its small specialty shops, which often source their products locally. In addition, Community-Supported Agriculture Programs have been gaining popularity in Italy, connecting consumers directly with local farmers (47). Participants in these programs pay in advance for a share of the farm's produce, providing financial support to the farmers while ensuring a steady supply of fresh, locally grown products for the consumers. Furthermore, Italy hosts numerous food festivals and fairs celebrating regional specialties and local produce. With the advancement of technology, many local farmers and producers in Italy have embraced e-commerce platforms to sell their products directly to consumers. This may allow for broader access to local food, even for those who may not have easy physical access to local markets or shops. All these aspects make the availability of local food a determining factor in its consumption.

Despite the abundance of local food options, challenges related to local food availability also exist in Italy. Among them, some local products are available only seasonally, limiting the year-round availability of certain items. In addition, the accessibility of local food in urban vs. rural areas is different. In urban areas, consumers often have greater access to various food retailers and markets, which may offer a wider selection of local products. However, the prices of local foods in urban settings might be influenced by higher operating costs and increased demand, potentially affecting affordability for some consumers. On the other hand, in rural areas, consumers might have more direct access to local farmers and producers through farmers' markets or farm-to-table initiatives, which can promote the consumption of locally sourced products. However, the variety and availability of local foods in rural settings could be limited compared to urban areas. Considering these factors, policymakers in Italy should continue to explore ways to enhance local food availability and accessibility. Our study showed that the availability of local food is a key factor, not so much when a food habit remains stable, but when people change their habits. Therefore, undertaking large-scale policies aimed at increasing access to and visibility of local food at points of sale, as well as information about its traceability, can be extremely important.

We have also observed that consumers perceive local food as less available when they describe their economic situation as low. This result can be better understood by considering what previous studies have found about the accessibility of healthy food (48). The easy access of high-income individuals to healthy food is linked to their proximity to supermarkets, which tend to stock a variety of healthy foods. In contrast, small independent grocery shops, which are often found in low-income neighborhoods, are less likely to have healthy options. This makes it harder for individuals living in low-income areas, who may also lack convenient transportation, to access healthy food. The same may be true for local food, but further studies are needed to confirm this. Policymakers should focus on understanding the logistical difficulties specific groups face in accessing local food and proposing solutions.

### 5.2. The effects of the intrinsic and extrinsic attributes of local food

According to previous studies showing the impact of taste on food preferences (10, 49), our study showed that taste is the most important predictor for evaluating the quality of local food, especially for consumers who have increased their consumption of local food. In addition, we found that authenticity was mostly important for people who did not yet buy local food (i.e., people who were in the absence stage). Our study also showed that healthiness and environmental respect were important consumers drivers, especially for those who reduced their consumption compared to that during the pandemic.

Social sustainability did not have a relevant weight in the evaluation of the quality of local food or in the purchase intention. This result is relevant because local and short-chain products can contribute to rural development and a sense of community by benefiting small local businesses beyond the market logic of wholesale (50). However, this prosocial attribute did not appear to influence consumer choice. This result is similar to what was observed in a pre–COVID-19 study, which found a stronger influence of self-centeredness than altruism in the context of local food consumption (10), as well as in a recent Italian study, which argues that local food is preferred by the consumer group that holds individualistic values (32). Considering this finding, the emerging relevance of environmental protection may thus be less related to an altruistic value and more to an interest in protecting the environment to ultimately control its impact on our health. To better investigate this tendency of Italian consumers, future studies could include other variables (such as moral and intrinsic motivations and social dominance orientation (51, 52) to assess how consumers' selfish and altruistic values influence the relevance associated with intrinsic and extrinsic attributes of local food.

Consistent with previous research (10), this study found that price expectations strongly influenced consumers' intention to buy local food. Consumers were less likely to choose local food when they were highly aware of the product's price, and the perception of local food as a high-quality product increased price expectations, which in turn reduced purchase intention. The impact of price expectations on purchase intention varies depending on the stage of consumer change toward buying local food, with price becoming important in both the absence and maintenance phases of the habit.

### 5.3. The impact of consumers' trust in local food producers

In our study, consumer trust in local food producers indirectly affected future intention to buy local food through perceived health, respect for the environment, and authenticity. In other words, trust in local food producers increased perceptions of local food as healthy, environmentally friendly, and authentic, and thus increased purchase intention. This result suggests that trust in the seller is likely to be related to an interaction with the consumer, which is why it can be called interpersonal trust (53). During a product purchase, the buyer and the seller communicate face-to-face about the production process, value, and concepts underlying the product, and interpersonal trust is thus fostered. Such communication provides consumers with information not only about the food producer but also about the product itself, and this information influences purchase intention (53). This reasoning confirms the importance of farmers' and urban markets, where citizens can engage directly with producers. Our findings also suggest that people with high levels of education trust local food producers more than people with low levels of education. Moreover, among highly educated people, the perception that local food respects the environment mediates the link between trust and the intention to buy local food. This suggests the importance of communication campaigns in the food sector (54) and the need to test the effectiveness of various types of content, especially based on the target audience they are intended for (55, 56).

It should be noted that, beyond the role of trust, price, availability, and quality, other crucial variables influence intention regarding local food. Therefore, any comprehensive analysis of local food consumption should consider these key determinants to gain a more accurate understanding of the factors that guide consumers toward choosing local food options. This is even more relevant when considering that these variables may have a direct effect on behavior beyond mere intention (57, 58). For example, if the price of local food is significantly higher than non-local options or if local products are not readily available in certain areas, consumers may opt for more affordable and easily accessible alternatives, even if they had the intention to buy local food.

### 5.4. Limitations

Our study has several limitations. First, given the existing gap between the intention to engage in a certain behavior and its actual execution, the lack of measurement of actual behavior is perhaps the most important limitation. Second, our questionnaire focused on local food in general, without specifying the different typologies of local agriculture and food categories. Third, we did not measure participants' ethnocentrism, which might have influenced their evaluation of local production. Fourth, in Italy, where the study was carried out, there is a specific definition of local food (e.g., zero kilometers and solidarity purchasing groups) and a specific availability of these products on the market (e.g., open-air markets and urban markets). On the one hand, by focusing on the unique aspects of Italian food culture, this study can offer valuable insights into the dynamics of local food consumption during the pandemic in this specific context. On the other hand, this detailed specificity might limit the direct transferability of the study's findings to other countries or regions with different food cultures and market structures. However, our research design paves the way for future research that compares our findings with what might emerge in other European and international contexts.

Finally, it is essential to recognize that our findings only represent the psychosocial perspective in a broader landscape of research on this subject. It is crucial to consider our results as part of a larger body of scientific knowledge that collectively sheds light on consumer behavior regarding local food. Future studies should build upon our research, incorporating multiple perspectives, including important economic factors. Including the evaluation of economic factors allows a better understanding of the impact of cost considerations on local food consumption. Exploring the economies of scale, pricing strategies, and accessibility of local food compared to non-local alternatives can provide a more comprehensive understanding of consumers' decision-making processes.

## 6. Conclusion

This research explores how consumers' perceptions and expectations of local food influence their intention to buy it, particularly in the context of the COVID-19 pandemic in Italy. The study identified three important factors that influence the intention to buy local food: availability, health, and trust in local food producers. The study suggests that promoting direct sales between producers and consumers could facilitate opportunities for interaction and increase the perception of local food as environmentally friendly, healthy, and authentic, thus promoting its consumption.

Based on our results, several food policy implications can be drawn to foster local food consumption and support sustainable food systems during the post-pandemic period. Given the central role of food availability in influencing consumers' intention to buy local food, policymakers should prioritize initiatives that increase access to and visibility of local food at various points of sale. Supporting farmers' open-air markets and urban markets can be an effective way to promote direct interactions between producers and consumers, fostering trust and facilitating communication about the production process and the value of local products. Encouraging large-scale policy interventions that facilitate the distribution of local food products to supermarkets and grocery stores can further enhance their accessibility. To tackle the issue of perceived low availability among economically disadvantaged consumers, policymakers should focus on understanding the logistical challenges faced by specific groups in accessing local food.

Our study also found that if local products are viewed as more expensive, the intent to purchase goes down. Implementing targeted support programs, such as subsidies or vouchers—especially for low-income individuals—to purchase local food, can help mitigate economic barriers and promote equitable access to locally sourced products. To address the impact of price expectations on local food purchase intention, policymakers can explore strategies to manage price perceptions. For instance, introducing price visibility labels on local food products or implementing price promotion initiatives can influence consumer perceptions of affordability and quality. Additionally, supporting local food producers in adopting cost-effective practices may help maintain competitive prices while preserving product quality.

Policymakers should collaborate with local food producers to emphasize the intrinsic attributes of local food, particularly taste and authenticity, as key drivers of consumer choices. Providing adequate and effective information on health benefits and environmental sustainability can enhance consumer awareness and appreciation of local food quality. In this vein, policymakers can support initiatives that foster trust in local food producers, recognizing the importance of interpersonal trust in influencing consumer behavior. Supporting farmers' markets and facilitating face-to-face interactions between producers and consumers can strengthen trust and establish lasting relationships. Promoting transparency and traceability in food production processes can also bolster consumer confidence in the authenticity and safety of local food products.

## Data availability statement

The raw data supporting the conclusions of this article will be made available by the authors, without undue reservation.

## Ethics statement

The studies involving humans were approved by Ethics Commission of the Catholic University of the Sacred Heart-Milan. The studies were conducted in accordance with the local legislation and institutional requirements. The participants provided their written informed consent to participate in this study.

## Author contributions

VC: conceptualization, methodology, visualization, resources, formal analyses, data curation, and writing—original draft. PC: conceptualization, methodology, and writing—original draft, supervision. All authors contributed to the article and approved the submitted version.
