# Supplementary material for: Psychosocial drivers influencing local food purchasing: beyond availability, the importance of trust in farmers
Source: Front Nutr. 2023 Sep 29;10:1204732. doi: 10.3389/fnut.2023.1204732 (PMC10580977; doi:10.3389/fnut.2023.1204732)
Supplement: Supplementary file 1 [file Data_Sheet_1.docx]

**Appendix**

**Appendix A** – **Table 1.** The goodness of fit and standardized coefficients for each nested model

|  | Model 1  (Local Food Attributes) | Model 2 (Local Food Attributes Plus Local Food Availability) | Model 3  (Model 2 Plus  Trust in Local Food Producers) | Model 4  (Model 3 Plus Control Variables) |
| --- | --- | --- | --- | --- |
| ***χ^2^*** (df) | 776.58 (73);  *p =* 0.001 | 562.13 (65);  *p =* 0.001 | 165.59 (54);  *p =* 0.001 | 13.76 (8);  *p =* 0.09 |
| RMSEA | 0.14 | 0.12 | 0.06 | 0.04 |
| CFI | 0.79 | 0.85 | 0.97 | 0.99 |
| TLI | 0.72 | 0.77 | 0.94 | 0.98 |
| SRMR | 0.19 | 0.15 | 0.06 | 0.05 |
| Trust → Appearance | Fixed to 0 | Fixed to 0 | 0.55* | 0.55* |
| Age → Appearance | Fixed to 0 | Fixed to 0 | Fixed to 0 | 0.01 |
| Sex → Appearance | Fixed to 0 | Fixed to 0 | Fixed to 0 | 0.05 |
| Education → Appearance | Fixed to 0 | Fixed to 0 | Fixed to 0 | –0.00 |
|  |  |  |  |  |
| Trust → Taste | Fixed to 0 | Fixed to 0 | 0.52* | 0.52* |
| Age → Taste | Fixed to 0 | Fixed to 0 | Fixed to 0 | 0.06 |
| Sex → Taste | Fixed to 0 | Fixed to 0 | Fixed to 0 | 0.05 |
| Education → Taste | Fixed to 0 | Fixed to 0 | Fixed to 0 | 0.05 |
|  |  |  |  |  |
| Trust → Authenticity | Fixed to 0 | Fixed to 0 | 0.58* | 0.58* |
| Age → Authenticity | Fixed to 0 | Fixed to 0 | Fixed to 0 | 0.02 |
| Sex → Authenticity | Fixed to 0 | Fixed to 0 | Fixed to 0 | 0.06 |
| Education → Authenticity | Fixed to 0 | Fixed to 0 | Fixed to 0 | 0.06 |
|  |  |  |  |  |
| Trust → Healthiness | Fixed to 0 | Fixed to 0 | 0.57* | 0.57* |
| Age → Healthiness | Fixed to 0 | Fixed to 0 | Fixed to 0 | 0.00 |
| Sex → Healthiness | Fixed to 0 | Fixed to 0 | Fixed to 0 | 0.06 |
| Education → Healthiness | Fixed to 0 | Fixed to 0 | Fixed to 0 | 0.02 |
|  |  |  |  |  |
| Trust → Environment Respect | Fixed to 0 | Fixed to 0 | 0.55* | 0.55* |
| Age → Environment Respect | Fixed to 0 | Fixed to 0 | Fixed to 0 | –0.04 |
| Sex → Environment Respect | Fixed to 0 | Fixed to 0 | Fixed to 0 | 0.05 |
| Education → Environment Respect | Fixed to 0 | Fixed to 0 | Fixed to 0 | 0.07 |
|  |  |  |  |  |
| Trust → Social Sustainability | Fixed to 0 | Fixed to 0 | 0.59* | 0.59* |
| Age → Social Sustainability | Fixed to 0 | Fixed to 0 | Fixed to 0 | 0.04 |
| Sex → Social Sustainability | Fixed to 0 | Fixed to 0 | Fixed to 0 | 0.01 |
| Education → Social Sustainability | Fixed to 0 | Fixed to 0 | Fixed to 0 | 0.00 |
|  |  |  |  |  |
| Appearance → Quality | 0.07* | 0.07* | 0.07* | 0.06* |
| Taste → Quality | 0.35* | 0.35* | 0.34* | 0.34* |
| Authenticity → Quality | 0.27* | 0.27* | 0.27* | 0.27* |
| Environment Respect → Quality | 0.09* | 0.09* | 0.09* | 0.09* |
| Healthiness → Quality | 0.17* | 0.17* | 0.17* | 0.17* |
| Social Sustainability → Quality | 0.02 | 0.02 | 0.01 | 0.01 |
| Age → Quality | Fixed to 0 | Fixed to 0 | Fixed to 0 | 0.00 |
| Sex → Quality | Fixed to 0 | Fixed to 0 | Fixed to 0 | 0.01 |
| Education → Quality | Fixed to 0 | Fixed to 0 | Fixed to 0 | –0.04 |
| Trust → Quality | Fixed to 0 | Fixed to 0 | 0.03 | 0.03 |
|  |  |  |  |  |
| Quality → Price | 0.22* | 0.22* | 0.13* | 0.13* |
| Trust → Price | Fixed to 0 | Fixed to 0 | 0.14* | 0.14* |
| Age → Price | Fixed to 0 | Fixed to 0 | Fixed to 0 | 0.00 |
| Sex → Price | Fixed to 0 | Fixed to 0 | Fixed to 0 | 0.13* |
| Education → Price | Fixed to 0 | Fixed to 0 | Fixed to 0 | 0.07 |
| Family Economic Condition → Price | Fixed to 0 | Fixed to 0 | Fixed to 0 | –0.00 |
|  |  |  |  |  |
| Age → Availability | Fixed to 0 | Fixed to 0 | Fixed to 0 | 0.02 |
| Sex → Availability | Fixed to 0 | Fixed to 0 | Fixed to 0 | –0.00 |
| Education → Availability | Fixed to 0 | Fixed to 0 | Fixed to 0 | –0.00 |
| Family Economic Condition → Availability | Fixed to 0 | Fixed to 0 | Fixed to 0 | 0.14* |
|  |  |  |  |  |
| Age → Trust | Fixed to 0 | Fixed to 0 | Fixed to 0 | 0.15 |
| Sex → Trust | Fixed to 0 | Fixed to 0 | Fixed to 0 | –0.00 |
| Education → Trust | Fixed to 0 | Fixed to 0 | Fixed to 0 | 0.62* |
|  |  |  |  |  |
| Appearance → Intention | –0.01 | –0.04 | –0.05 | –0.05 |
| Taste → Intention | 0.09 | 0.05 | 0.05 | 0.05 |
| Authenticity → Intention | 0.14* | 0.10 | 0.10 | 0.10 |
| Environment Respect → Intention | 0.16* | 0.16* | 0.16* | 0.16* |
| Healthiness → Intention | 0.15* | 0.16* | 0.15* | 0.15* |
| Social Sustainability → Intention | 0.06 | 0.03 | 0.02 | 0.02 |
| Quality → Intention | 0.15* | 0.13* | 0.13* | 0.13* |
| Price → Intention | –0.10* | –0.10* | –0.10* | –0.10* |
| Availability → Intention | Fixed to 0 | 0.21* | 0.20* | 0.20* |
| Trust → Intention | Fixed to 0 | Fixed to 0 | 0.04 | 0.05 |
| Age → Intention | Fixed to 0 | Fixed to 0 | Fixed to 0 | 0.09* |
| Sex → Intention | Fixed to 0 | Fixed to 0 | Fixed to 0 | –0.00 |
| Education → Intention | Fixed to 0 | Fixed to 0 | Fixed to 0 | 0.08* |
| Family Economic Condition → Intention | Fixed to 0 | Fixed to 0 | Fixed to 0 | –0.04 |
|  |  |  |  |  |
| Appearance → Quality → Intention | 0.01 | 0.01 | 0.01 | 0.01 |
| Taste → Quality → Intention | 0.04* | 0.04* | 0.04* | 0.04* |
| Authenticity → Quality → Intention | 0.05* | 0.05* | 0.05* | 0.05* |
| Healthiness → Quality → Intention | 0.02* | 0.02* | 0.02* | 0.02* |
| Environment Respect→ Quality → Intention | 0.01 | 0.01 | 0.01 | 0.01 |
| Trust → Quality → Intention | 0.01 | 0.01 | 0.01 | 0.01 |
|  |  |  |  |  |
| Family Economic Condition → Availability → Intention | Fixed to 0 | Fixed to 0 | Fixed to 0 | –0.03* |
|  |  |  |  |  |
| Quality → Price → Intention | –0.01* | –0.01* | –0.01* | –0.01* |
| Trust → Price → Intention | Fixed to 0 | Fixed to 0 | –0.01* | –0.01* |
| Sex → Price → Intention | Fixed to 0 | Fixed to 0 | Fixed to 0 | –0.01* |
|  |  |  |  |  |
| Appearance → Quality → Price → Intention | –0.00 | –0.00 | –0.00 | –0.00 |
| Taste → Quality → Price → Intention | –0.01* | –0.01* | –0.01* | –0.01* |
| Authenticity → Quality → Price → Intention | –0.01* | –0.01* | –0.01* | –0.01* |
| Healthiness → Quality → Price → Intention | –0.00 | –0.00 | –0.00 | –0.00 |
| Environment Respect → Quality → Price → Intention | –0.00 | –0.00 | –0.00 | –0.00 |
|  |  |  |  |  |
|  |  |  |  |  |
|  |  |  |  |  |
| Education → Trust → Appearance → Intention | Fixed to 0 | Fixed to 0 | Fixed to 0 | –0.01 |
| Education → Trust → Taste → Intention | Fixed to 0 | Fixed to 0 | Fixed to 0 | 0.01 |
| Education → Trust → Authenticity → Intention | Fixed to 0 | Fixed to 0 | Fixed to 0 | 0.04 |
| Education → Trust → Environment Respect → Intention | Fixed to 0 | Fixed to 0 | Fixed to 0 | 0.05* |
| Education → Trust → Healthiness → Intention | Fixed to 0 | Fixed to 0 | Fixed to 0 | 0.06* |
| Education → Trust → Social Sustainability → Intention | Fixed to 0 | Fixed to 0 | Fixed to 0 | 0.00 |
| Education → Trust → Quality → Intention | Fixed to 0 | Fixed to 0 | Fixed to 0 | 0.00 |
| Education → Trust → Price → Intention | Fixed to 0 | Fixed to 0 | Fixed to 0 | –0.01 |
|  |  |  |  |  |
|  |  |  |  |  |
| Trust → Authenticity → Intention | Fixed to 0 | Fixed to 0 | 0.06 | 0.06 |
| Trust → Healthiness → Intention | Fixed to 0 | Fixed to 0 | 0.09* | 0.09* |
| Trust → Environment Respect → Intention | Fixed to 0 | Fixed to 0 | 0.08* | 0.08* |
| Trust → Appearance → Quality → Intention | Fixed to 0 | Fixed to 0 | 0.00 | 0.00 |
| Trust → Taste → Quality → Intention | Fixed to 0 | Fixed to 0 | 0.02* | 0.02* |
| Trust → Authenticity → Quality → Intention | Fixed to 0 | Fixed to 0 | 0.03* | 0.03* |
| Trust → Healthiness → Quality → Intention | Fixed to 0 | Fixed to 0 | 0.01 | 0.01 |
| Trust → Quality → Price → Intention | Fixed to 0 | Fixed to 0 | 0.00 | 0.00 |
| Trust → Appearance → Quality → Price → Intention | Fixed to 0 | Fixed to 0 | –0.01 | –0.01 |
| Trust → Taste → Quality → Price → Intention | Fixed to 0 | Fixed to 0 | –0.01 | –0.01 |
| Trust → Authenticity → Quality → Price → Intention | Fixed to 0 | Fixed to 0 | –0.01* | –0.01* |
| Trust → Healthiness → Quality → Price → Intention | Fixed to 0 | Fixed to 0 | –0.01 | –0.01 |
| Trust → Environment Respect → Quality → Price → Intention | Fixed to 0 | Fixed to 0 | –0.01 | –0.01 |
|  |  |  |  |  |
| *R*^2^ Quality | 0.70** | 0.70** | 0.70** | 0.70* |
| *R*^2^ Price | 0.05** | 0.05** | 0.06** | 0.08* |
| *R^2^* Intention | 0.40** | 0.42** | 0.42** | 0.44* |

Note: **p* < 0.001; *χ2* = goodness-of-fit statistics, *df* = degrees of freedom of chi-square statistics, CFI = Comparative Fit Index, TLI = Tucker-Lewis fit Index; RMSEA = Root Mean Square Error of Approximation

**Appendix A** – **Table 2.** Results of the comparisons of the direct paths among participants’ stages of change

|  |  | Absence Stage  versus  Reduction Stage | Absence Stage  versus  Maintenance Stage | Absence Stage  versus  Increase Stage | Reduction Stage  versus  Maintenance Stage | Reduction Stage  versus  Increase Stage | Maintenance Stage  versus  Increase Stage |
| --- | --- | --- | --- | --- | --- | --- | --- |
| a | Appearance →  Future Intention | χ2(1) = 3.78 *p* = 0.05 | χ2(1) = 0.20 *p* = 0.65 | χ2(1) = 0.78 *p* = 0.38 | χ2(1) = 3.80 *p* = 0.05 | χ2(1) = 1.61 *p* = 0.20 | *χ*^2^(1) = 0.44 *p* = 0.51 |
| b | Taste →  Quality | *χ*^2^(1) = 0.18 *p* = 0.67 | *χ*^2^(1) = 2.27 *p* = 0.13 | *χ*^2^(1) = 5.90 *p* = 0.01 | *χ*^2^(1) = 0.18 *p* = 0.67 | *χ*^2^(1) = 4.10 *p* = 0.05 | *χ*^2^(1) = 14.14 *p* = 0.01 |
| c | Authenticity→  Quality | *χ*^2^(1) = 7.37 *p* = 0.01 | χ2(1) = 0.31 p = 0.58 | χ2(1) = 9.31 p = 0.01 | *χ*^2^(1) = 5.54 *p* = 0.02 | *χ*^2^(1) = 20.24 *p* = 0.001 | *χ*^2^(1) = 11.40 *p* = 0.01 |
| d | Authenticity→  Future Intention | χ2(1) = 0.56 *p* = 0.45 | χ2(1) = 2.90, *p* = 0.09 | χ2(1) = 0.34 *p* = 0.55 | χ2(1) = 0.35 *p* = 0.55 | χ2(1) = 0.08 *p* = 0.77 | χ2(1) = 1.40 *p* = 0.24 |
| e | Environment Respect →  Quality | χ2(1) = 2.30 *p* = 0.13 | χ2(1) = 2.62 *p* = 0.11 | χ2(1) = 0.11 *p* = 0.74 | χ2(1) = 0.64 *p* = 0.42 | χ2(1) = 1.48 *p*= 0.22 | χ2(1) = 0.74 *p* = 0.39 |
| f | Environment Respect →  Future Intention | χ2(1) = 0.00 *p* = 0.96 | χ2(1) = 0.00 *p* = 0.94 | χ2(1) = 0.01 *p* = 0.92 | χ2(1) = 0.00 *p* = 0.99 | χ2(1) = 0.02 *p* = 0.89 | χ2(1) = 0.05 *p* = 0.82 |
| g | Healthiness→  Quality | χ2(1) = 5.79 *p* = 0.02 | χ2(1) = 0.38 *p* = 0.53 | χ2(1) = 0.03 *p* = 0.87 | χ2(1) = 7.99 *p* = 0.001 | χ2(1) = 5.29 *p* = 0.02 | χ2(1) = 0.11 *p* = 0.74 |
| h | Healthiness →  Future Intention | χ2(1) = 2.48 *p* = 0.11 | χ2(1) = 0.41 *p* = 0.52 | χ2(1) = 0.10 *p* = 0.75 | χ2(1) = 6.40 *p* = 0.01 | χ2(1) = 4.58 *p* = 0.03 | χ2(1) = 0.14  *p* = 0.71 |
| i | Quality →  Future Intention | χ2(1) = 1.37 *p* = 0.24 | χ2(1) = 2.52 *p* = 0.11 | χ2(1) = 0.94, *p* = 0.33 | χ2(1) = 0.15 *p* = 0.69 | χ2(1) = 0.09 *p* = 0.76 | χ2(1) = 0.62 *p* = 0.43 |
| j | Quality →  Price | χ2(1) = 0.35 *p* = 0.55 | χ2(1) = 0.20 *p* = 0.65 | χ2(1) = 2.60 *p* = 0.11 | χ2(1) = 0.08 *p* = 0.77 | χ2(1) = 4.57 *p* = 0.03 | χ2(1) = 6.21 *p* = 0.01 |
| k | Price→  Future Intention | χ2(1) = 8.36 *p* = 0.01 | χ2(1) = 0.33 *p* = 0.56 | χ2(1) = 5.79 *p* = 0.02 | χ2(1) = 7.88 *p*= 0.01 | χ2(1) = 0.93 *p* = 0.33 | χ2(1) = 5.39 *p* = 0.02 |
| l | Local Food Availability→  Future Intention | χ2(1) = 1.70 *p* = 0.19 | χ2(1) = 0.68 *p*= 0.40 | χ2(1) = 4.11 *p* = 0.04 | χ2(1) = 0.45 *p* = 0.50 | χ2(1) = 0.39 *p* = 0.53 | χ2(1) = 2.15 *p* = 0.14 |
| m | Trust in Local Food  Producers→  Appearance | *χ*^2^(1) = 1.71 *p* = 0.19 | *χ*^2^(1) = 0.09 *p* = 0.78 | *χ*^2^(1) = 0.64 *p* = 0.42 | *χ*^2^(1) = 2.39 *p* = 0.12 | *χ*^2^(1) = 0.35  *p* = 0.55 | *χ*^2^(1) = 0.35  *p* = 0.55 |
| n | Trust in Local Food Producers→  Taste | *χ*^2^(1) = 16.87 *p* = 0.001 | *χ*^2^(1) = 4.48 *p* = 0.04 | *χ*^2^(1) = 0.00 *p* = 0.99 | *χ*^2^(1) = 8.31 *p* = 0.001 | *χ*^2^(1) = 15.19 *p* = 0.001 | *χ*^2^(1) = 3.96 *p* = 0.05 |
| o | Trust in Local Food Producers→  Authenticity | *χ*^2^(1) = 0.24 *p* = 0.62 | *χ*^2^(1) = 0.01 *p* = 0.92 | *χ*^2^(1) = 0.04 *p* = 0.84 | *χ*^2^(1) = 0.24 *p* = 0.62 | *χ*^2^(1) = 0.52  *p* = 0.47 | *χ*^2^(1) = 0.14 *p* = 0.70 |
| p | Trust in Local Food  Producers→  Environment Respect | *χ*^2^(1) = 3.08 *p* = 0.08 | *χ*^2^(1) = 2.14 *p* = 0.14 | *χ*^2^(1) = 2.84 *p* = 0.09 | *χ*^2^(1) = 0.46 *p* = 0.50 | *χ*^2^(1) = 0.07 *p* = 0.79 | *χ*^2^(1) = 0.21 *p* = 0.65 |
| q | Trust in Local Food  Producers→  Healthiness | *χ*^2^(1) = 7.96 *p* = 0.01 | *χ*^2^(1) = 0.60 *p* = 0.44 | *χ*^2^(1) = 0.03 *p* = 0.85 | *χ*^2^(1) = 6.15, *p* = 0.01 | *χ*^2^(1) = 7.09 *p* = 0.01 | *χ*^2^(1) = 0.29 *p* = 0.59 |
| r | Trust in Local Food  Producers→  Social Sustainability | *χ*^2^(1) = 0.12 *p* = 0.72 | *χ*^2^(1) = 3.60 *p* = 0.05 | *χ*^2^(1) = 6.67 *p* = 0.01 | *χ*^2^(1) = 1.81 *p* = 0.17 | *χ*^2^(1) = 4.36  *p* = 0.04 | *χ*^2^(1) = 1.33 *p* = 0.25 |
| s | Trust in Local Food  Producers→  Price | *χ*^2^(1) = 3.67 *p* = 0.05 | *χ*^2^(1) = 8.71 *p* = 0.01 | *χ*^2^(1) = 3.19 *p* = 0.07 | *χ*^2^(1) = 0.27 *p* = 0.60 | *χ*^2^(1) = 0.06 *p* = 0.80 | *χ*^2^(1) = 0.88 *p* = 0.35 |
| t | Sex →  Authenticity | *χ*^2^(1) = 0.09  *p* = 0.76 | *χ*^2^(1) = 1.14  *p* = 0.28 | *χ*^2^(1) = 1.11  *p* = 0.29 | *χ*^2^(1) = 1.11  *p* = 0.29 | *χ*^2^(1) = 0.18  *p* = 0.67 | *χ*^2^(1) = 0.05  *p* = 0.81 |
| u | Sex →  Environment Respect | *χ*^2^(1) = 1.56  *p* = 0.21 | *χ*^2^(1) = 3.75  *p* = 0.05 | *χ*^2^(1) = 0.48  *p* = 0.49 | *χ*^2^(1) = 0.00  *p* = 0.99 | *χ*^2^(1) = 0.32  *p* = 0.56 | *χ*^2^(1) = 0.62  *p* = 0.43 |
| v | Sex →  Trust in Local Food Producers | *χ*^2^(1) = 0.67  *p* = 0.41 | *χ*^2^(1) = 3.76  *p* = 0.05 | *χ*^2^(1) = 0.60  *p* = 0.43 | *χ*^2^(1) = 0.04  *p* = 0.84 | *χ*^2^(1) = 0.12  *p* = 0.72 | *χ*^2^(1) = 0.23  *p* = 0.63 |
| w | Sex →  Quality | *χ*^2^(1) = 3.99  *p* = 0.04 | *χ*^2^(1) = 0.20  *p* = 0.65 | *χ*^2^(1) = 0.00  *p* = 0.98 | *χ*^2^(1) = 3.75  *p* = 0.05 | *χ*^2^(1) = 3.76  *p* = 0.05 | *χ*^2^(1) = 0.11  *p* = 0.73 |
| x | Sex →  Price | *χ*^2^(1) = 2.26  *p* = 0.13 | *χ*^2^(1) = 0.49  *p* = 0.48 | *χ*^2^(1) = 0.02  *p* = 0.87 | *χ*^2^(1) = 4.31  *p* = 0.04 | *χ*^2^(1) = 1.16  *p* = 0.20 | *χ*^2^(1) = 0.61  *p* = 0.43 |
| y | Education →  Trust in Local Food Producers | *χ*^2^(1) = 0.84  *p* = 0.36 | *χ*^2^(1) = 1.89  *p* = 0.17 | *χ*^2^(1) = 0.08  *p* = 0.77 | *χ*^2^(1) = 0.10  *p* = 0.74 | *χ*^2^(1) = 0.04  *p* = 0.82 | *χ*^2^(1) = 0.05  *p* = 0.81 |
| z | Education →  Price | *χ*^2^(1) = 0.35  *p* = 0.55 | *χ*^2^(1) = 0.62  *p* = 0.43 | *χ*^2^(1) = 4.07  *p* = 0.04 | *χ*^2^(1) = 0.01  *p* = 0.93 | *χ*^2^(1) = 4.76  *p* = 0.03 | *χ*^2^(1) = 8.54  *p* = 0.01 |
| aa | Age →  Quality | *χ*^2^(1) = 10.52  *p* = 0.01 | *χ*^2^(1) = 8.6,  *p* = 0.01 | *χ*^2^(1) = 6.09 *p* = 0.01 | *χ*^2^(1) = 2.87 *p* = 0.09 | *χ*^2^(1) = 1.26  *p* = 0.26 | *χ*^2^(1) = 0.24  *p* = 0.62 |
| ab | Age →  Environment Respect | *χ*^2^(1) = 1.91  *p* = 0.16 | *χ*^2^(1) = 2.55  *p* = 0.11 | *χ*^2^(1) = 2.39 *p* = 0.12 | *χ*^2^(1) = 0.11 *p* = 0.75 | *χ*^2^(1) = 0.00  *p* = 0.98 | *χ*^2^(1) = 0.13  *p* = 0.71 |
| ac | Age →  Future Intention | *χ*^2^(1) = 0.00 *p* = 0.98 | *χ*^2^(1) = 0.82  *p* = 0.36 | *χ*^2^(1) = 0.90  *p* = 0.34 | *χ*^2^(1) = 0.63  *p* = 0.42 | *χ*^2^(1) = 0.73  *p* = 0.39 | *χ*^2^(1) = 0.04  *p* = 0.85 |
| ad | Age →  Local Food Availability | *χ*^2^(1) = 1.80  *p* = 0.18 | *χ*^2^(1) = 0.59  *p* = 0.44 | *χ*^2^(1) = 4.70  *p* = 0.03 | *χ*^2^(1) = 0.87  *p* = 0.35 | *χ*^2^(1) = 0.17  *p* = 0.67 | *χ*^2^(1) = 3.24  *p* = 0.07 |
| ae | Family Economic  Condition →  Price | *χ*^2^(1) = 1.49 *p* = 0.22 | *χ*^2^(1) = 4.11 *p* = 0.04 | *χ*^2^(1) = 6.31 *p* = 0.01 | *χ*^2^(1) = 1.04 *p* = 0.85 | *χ*^2^(1) = 0.99  *p* = 0.32 | *χ*^2^(1) = 1.19 *p* = 0.27 |
| af | Family Economic  Condition →  Future Intention | *χ*^2^(1) = 0.00  *p* = 0.94 | *χ*^2^(1) = 0.38 *p* = 0.53 | *χ*^2^(1) = 0.02 *p* = 0.87 | *χ*^2^(1) = 0.24 *p* = 0.62 | *χ*^2^(1) = 0.05  *p* = 0.82 | *χ*^2^(1) = 0.63 *p* = 0.42 |
| ag | Family Economic  Condition →  Local Food Availability | *χ*^2^(1) = 0.47 *p* = 0.49 | *χ*^2^(1) = 1.04 *p* = 0.31 | *χ*^2^(1) = 0.52 *p* = 0.47 | *χ*^2^(1) = 2.44  *p* = 0.12 | *χ*^2^(1) = 0.00  *p* = 0.99 | *χ*^2^(1) = 2.74  *p* = 0.09 |

**Appendix A** – **Table 3.** Results of the comparisons of the indirect paths among participants’ stages of change

|  |  | Absence Stage  versus  Reduction Stage | Absence Stage  versus  Maintenance Stage | Absence Stage  versus  Increase Stage | Reduction Stage  versus  Maintenance Stage | Reduction Stage  versus  Increase Stage | Maintenance Stage  versus  Increase Stage |
| --- | --- | --- | --- | --- | --- | --- | --- |
| a | Taste →  Quality →  Intention to Buy Local Food | *χ*^2^(1) = 0.92, *p* = 0.34 | *χ*^2^(1) = 1.18, *p* = 0.28 | *χ*^2^(1) = 1.39, *p* = 0.24 | *χ*^2^(1) = 0.00, *p* = 0.99 | *χ*^2^(1) = 0.01,  *p* = 0.92 | *χ*^2^(1) = 0.01, *p* = 0.90 |
| b | Authenticity→  Quality →  Intention to Buy Local Food | *χ*^2^(1) = 1.80,  *p* = 0.17 | *χ*^2^(1) = 2.49,  *p* = 0.11 | *χ*^2^(1) = 0.09,  *p* = 0.77 | *χ*^2^(1) = 0.16,  *p* = 0.69 | *χ*^2^(1) = 1.91,  *p* = 0.16 | *χ*^2^(1) = 5.78,  *p* = 0.02 |
| c | Healthiness→  Quality →  Intention to Buy Local Food | *χ*^2^(1) = 0.03, *p* = 0.85 | *χ*^2^(1) = 2.62, *p* = 0.10 | *χ*^2^(1) = 0.58, *p* = 0.44 | *χ*^2^(1) = 4.59, *p* = 0.03 | *χ*^2^(1) = 1.28,  *p* = 0.26 | *χ*^2^(1) = 1.10, *p* = 0.29 |
| d | Trust in Local Food Producers→  Appearance →  Intention to Buy Local Food | *χ*^2^(1) = 2.11,  *p* = 0.14 | *χ*^2^(1) = 0.15,  *p* = 0.69 | *χ*^2^(1) = 0.66,  *p* = 0.41 | *χ*^2^(1) = 2.21,  *p* = 0.14 | *χ*^2^(1) = 0.93,  *p* = 0.33 | *χ*^2^(1) = 0.39,  *p* = 0.53 |
| e | Trust in Local Food Producers→  Authenticity→  Intention to Buy Local Food | *χ*^2^(1) = 0.61, *p* = 0.44 | *χ^2^*(1) = 2.62,  *p* = 0.10 | *χ*^2^(1) = 0.24,  *p* = 0.65 | *χ*^2^(1) = 0.47,  *p* = 0.49 | *χ*^2^(1) = 0.14,  *p* = 0.71 | *χ*^2^(1) = 1.46,  *p* = 0.23 |
| f | Trust in Local Food Producers→  Environment Respect →  Intention to Buy Local Food | *χ*^2^(1) = 0.05, *p* = 0.83 | *χ*^2^(1) = 0.02,  *p* = 0.88 | *χ*^2^(1) = 0.12,  *p* = 0.73 | *χ*^2^(1) = 0.01,  *p* = 0.89 | *χ*^2^(1) = 0.01,  *p* = 0.92 | *χ*^2^(1) = 0.11,  *p* = 0.74 |
| g | Trust in Local Food Producers→  Authenticity→  Quality →  Intention to Buy Local Food | *χ*^2^(1) = 1.64,  *p* = 0.20 | *χ*^2^(1) = 2.40, *p* = 0.12 | *χ*^2^(1) = 0.08, *p* = 0.77 | *χ*^2^(1) = 0.08,  *p* = 0.78 | *χ*^2^(1) = 1.79,  *p* = 0.18 | *χ*^2^(1) = 5.46,  *p* = 0.02 |
| h | Trust in Local Food Producers→  Healthiness→  Quality →  Intention to Buy Local Food | *χ*^2^(1) = 0.03, *p* = 0.86 | *χ*^2^(1) = 2.30, *p* = 0.13 | *χ*^2^(1) = 0.57, *p* = 0.45 | *χ*^2^(1) = 5.20, *p* = 0.03 | *χ*^2^(1) = 0.95, *p* = 0.33 | *χ*^2^(1) = 0.89, *p* = 0.35 |
| i | Age →  Local Food Availability→  Intention to Buy Local Food | *χ*^2^(1) = 1.35, *p* = 0.24 | *χ*^2^(1) = 0.64, *p* = 0.42 | *χ*^2^(1) = 4.13, *p* = 0.04 | *χ*^2^(1) = 0.88, *p* = 0.35 | *χ*^2^(1) = 0.50, *p* = 0.48 | *χ*^2^(1) = 3.28, *p* = 0.07 |
| j | Age →  Local Food Availability→  Intention to Buy Local Food | *χ*^2^(1) = 151, *p* = 0.22 | *χ*^2^(1) =0.03, *p* = 0.87 | *χ*^2^(1) = 2.38, *p* = 0.12 | *χ*^2^(1) = 1.17, *p* = 0.18 | *χ*^2^(1) = 0.11, *p* = 0.74 | *χ*^2^(1) = 2.70, *p* = 0.10 |

**Appendix A** – **Figure 1.** Hypothesised integrated model to explain the consumers’ intention to buy local food after the COVID-19 pandemic.


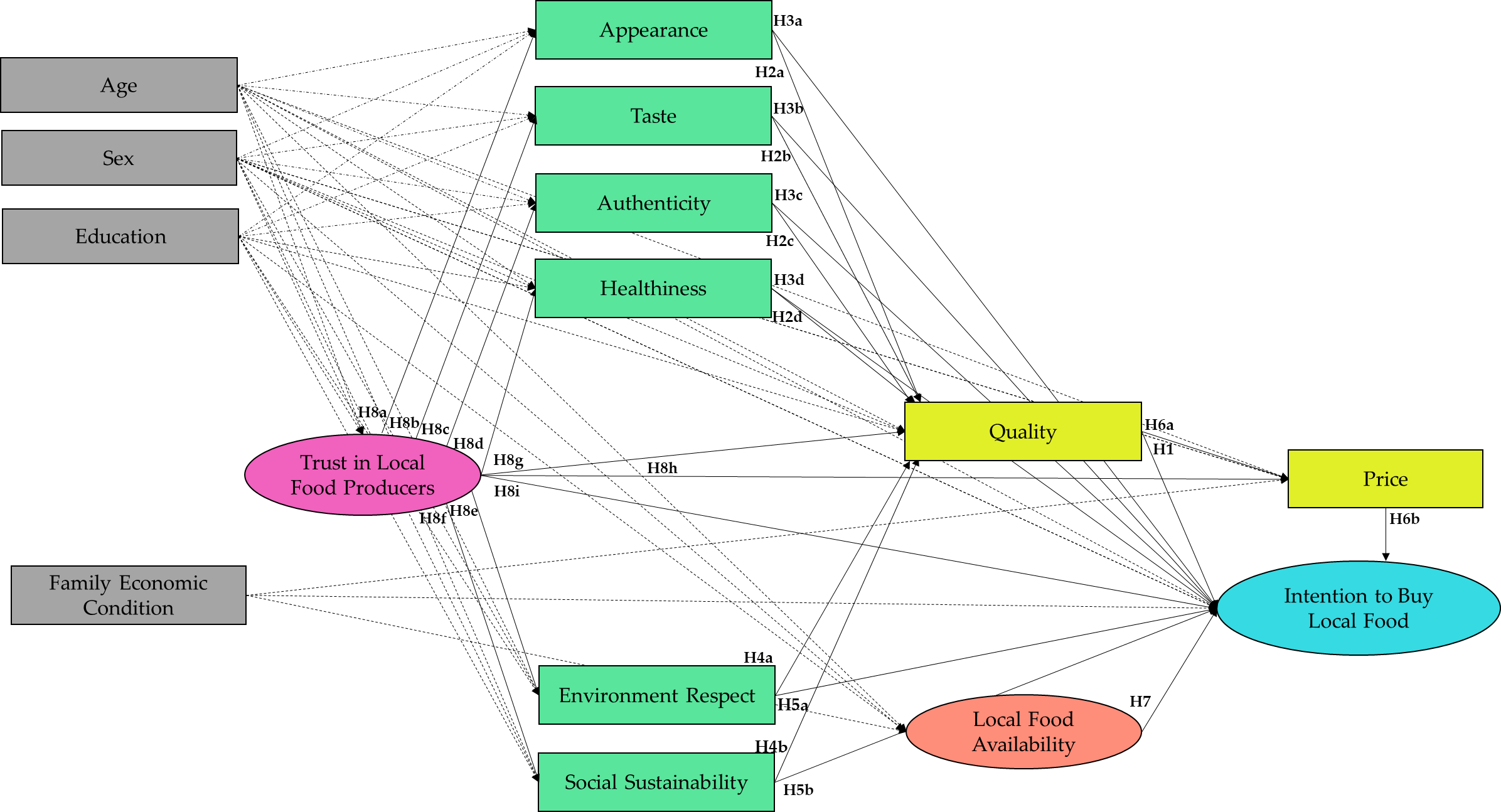


*Note.* Dashed arrows indicate research questions (RQ1 and RQ2). H = Hypothesis

**Appendix A** – **Figure 2.** Absence stage: results of the integrated model to explain the consumers’ intention to buy local food after COVID-19


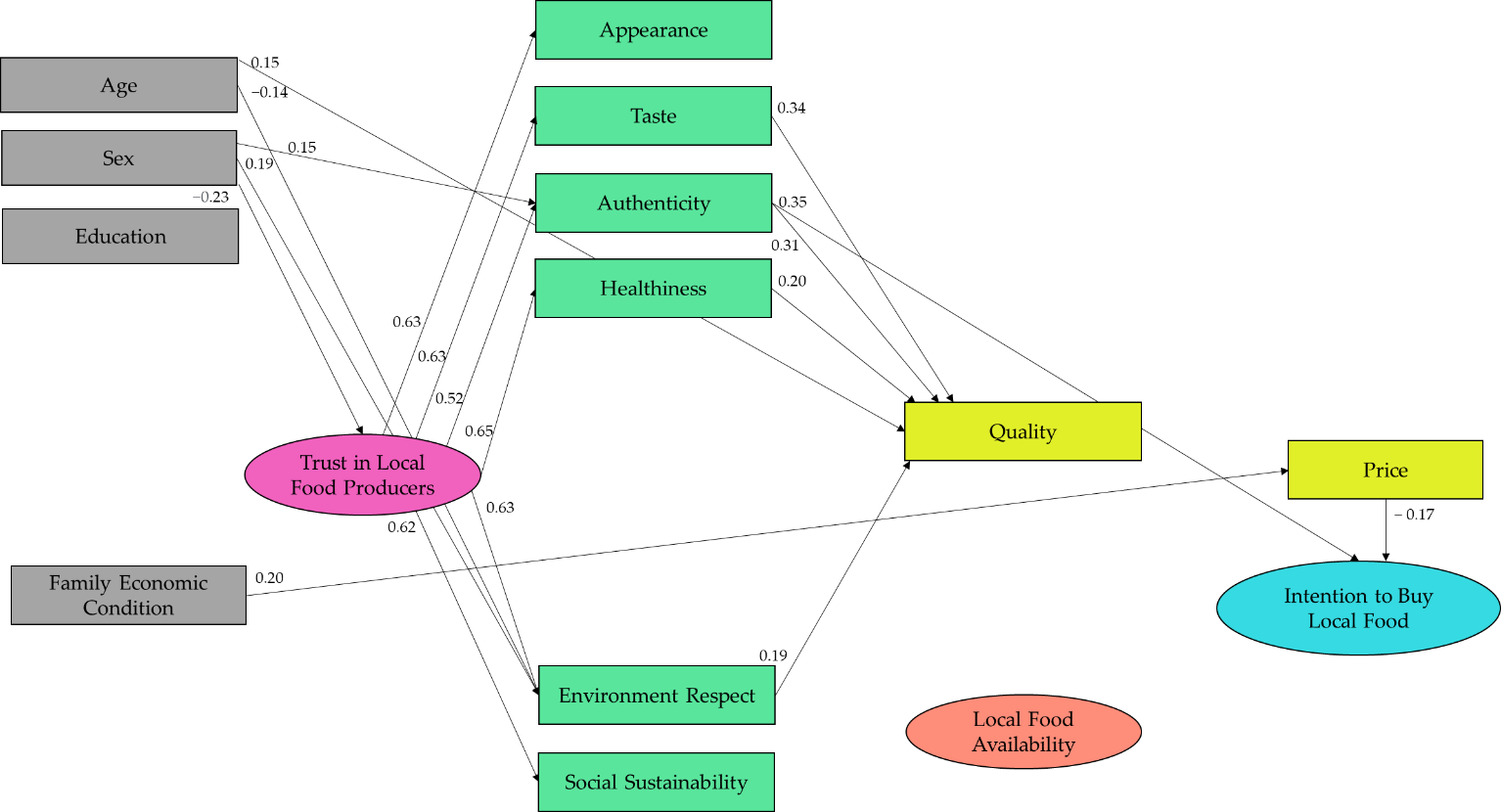


**Figure 3.** Reduction stage: results of the integrated model to explain the consumers’ intention to

buy local food after COVID-19

*
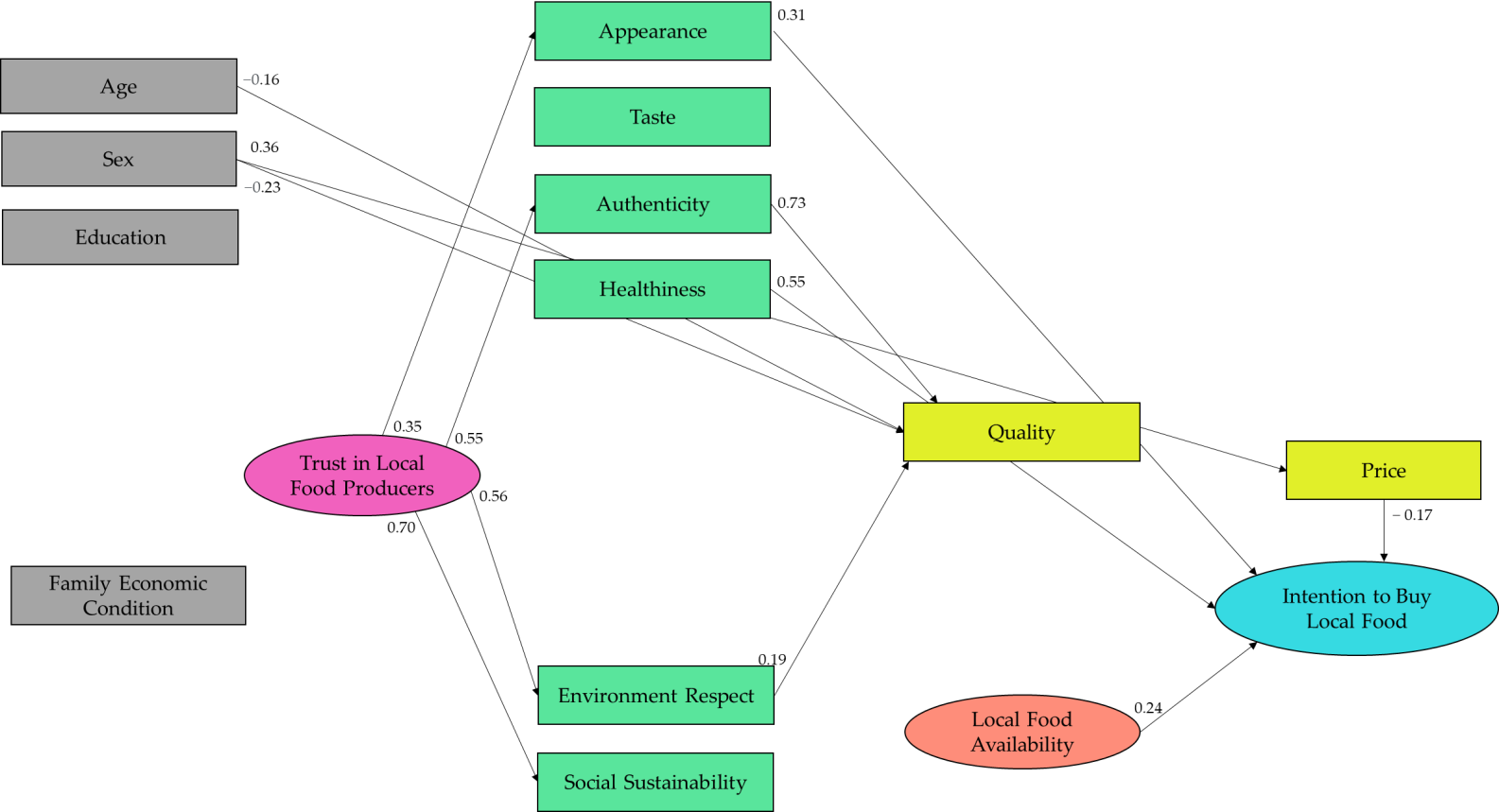
*

**Appendix A** – **Figure 4.** Maintenance stage: results of the integrated model to explain the consumers’ intention to buy local food after COVID-19


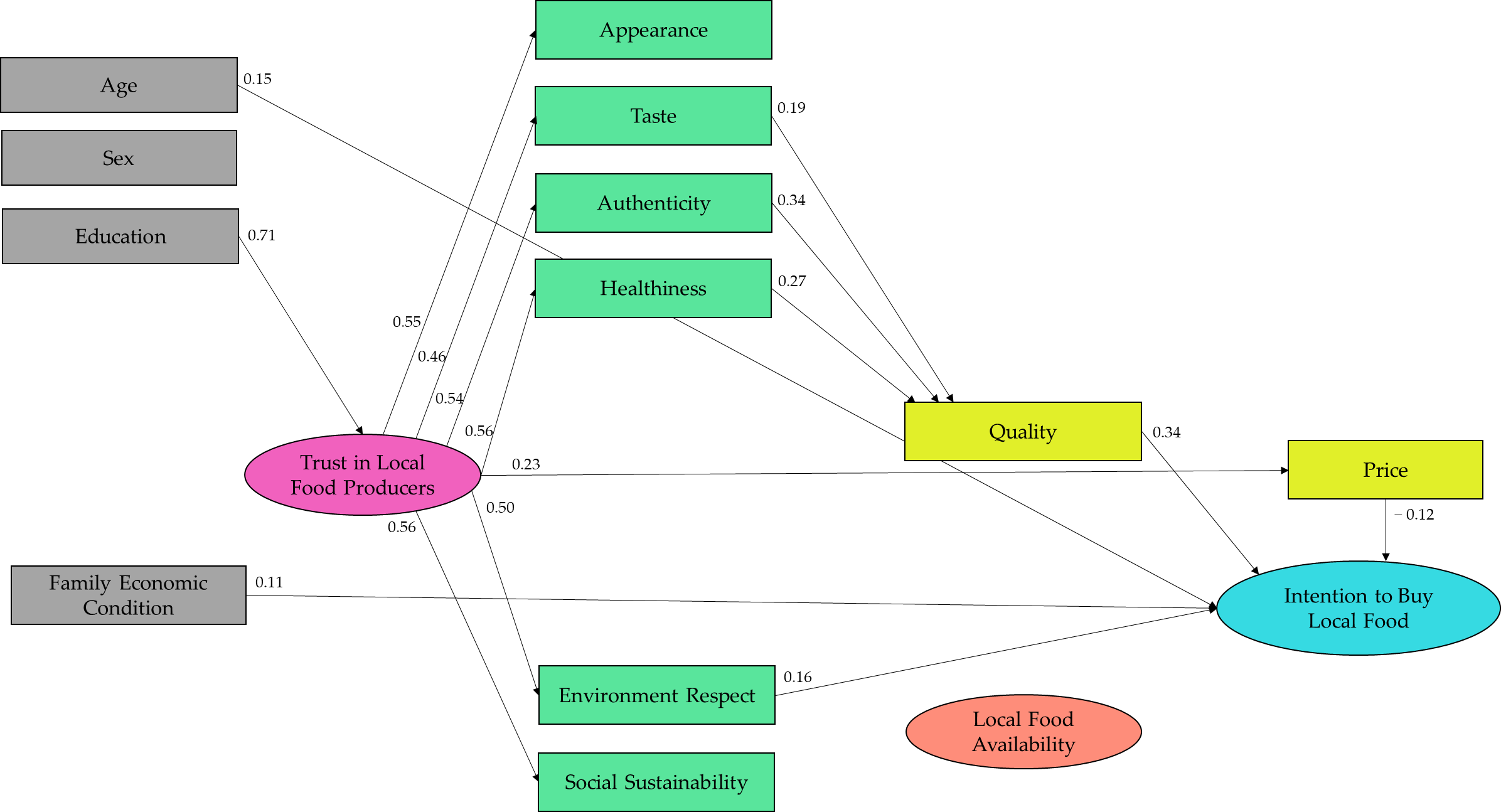


**Appendix A** – **Figure 5.** Increase stage: results of the integrated model to explain the consumers’ intention to buy local food after Covid-19

**
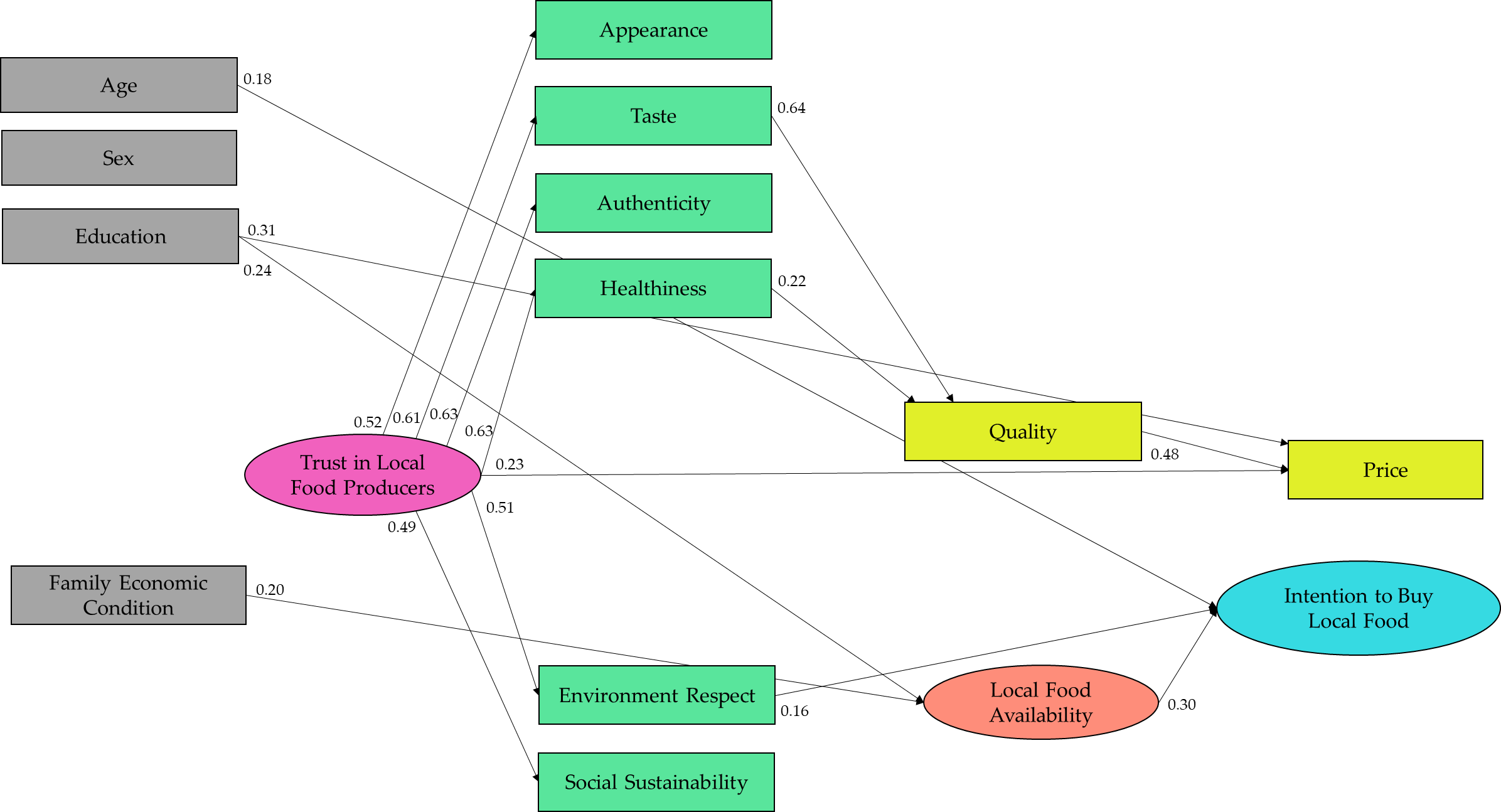
**
